# Senescent Tissue-Resident Mesenchymal Stromal Cells Are an Internal Source of Inflammation in Human Osteoarthritic Cartilage

**DOI:** 10.3389/fcell.2021.725071

**Published:** 2021-09-06

**Authors:** Wenguang Liu, Alexander S. Brodsky, Meng Feng, Yajun Liu, Jing Ding, Chathuraka T. Jayasuriya, Qian Chen

**Affiliations:** ^1^Department of Orthopedics, Rhode Island Hospital, Alpert Medical School of Brown University, Providence, RI, United States; ^2^Department of Pathology and Laboratory Medicine, Rhode Island Hospital, Alpert Medical School of Brown University, Providence, RI, United States; ^3^Center for Computational Molecular Biology, Brown University, Providence, RI, United States

**Keywords:** osteoarthritis, mesenchymal stem cell, cell senescence, cartilage, SASP

## Abstract

Human osteoarthritic cartilage contains not only chondrocytes (OACs), but also mesenchymal stromal cells (OA-MSCs), whose abundance increases during osteoarthritis (OA). However, it is not clear how OA-MSC contributes to OA pathogenesis. Here, we show that aging OA-MSC plays an important role in cell senescence, fibrosis, and inflammation in cartilage. Protein array analysis indicates that OA-MSC expresses pro-inflammatory senescence associated secretory phenotype (SASP) including IL-1β, IL-6, IL-8, and CXCL1, 5, and 6, which play key roles in OA pathogenesis. OAC is a main recipient of the inflammatory signals by expressing receptors of cytokines. RNAseq analysis indicates that the transition from normal cartilage stromal cells (NCSCs) to OA-MSC during aging results in activation of SASP gene expression. This cell transition process can be recapitulated by a serial passage of primary OAC in cell culture comprising (1) OAC dedifferentiation into NCSC-like cells, and (2) its subsequent senescence into pro-inflammatory OA-MSC. While OAC dedifferentiation is mediated by transcriptional repression of chondrogenic gene expression, OA-MSC senescence is mediated by transcriptional activation of SASP gene expression. We postulate that, through replication-driven OAC dedifferentiation and mesenchymal stromal cell (MSC) senescence, OA-MSC becomes an internal source of sterile inflammation in human cartilage joint.

## Introduction

Osteoarthritis (OA) is an aging associated joint degenerativedisease affecting more than 300 million people worldwide. It is characterized by cartilage degradation, bone remodeling, and low-grade inflammation termed “inflammaging” (Coryell et al., 2020). However, the pathological mechanism of OA has not been well understood, which contributes to the lack of any FDA-approved drugs to modify OA pathogenesis. Although OA was not considered as an inflammatory disease historically, it has become more evident in recent years that the persistent long-term elevation of low-grade inflammation plays an important role in OA pathogenesis (Robinson et al., 2016). The sustained chronic production/presence of cytokines including TNF-α, IL-1β, IL-6, IL-8, and IL-17 and chemokines including CXCL1, 3, 5, and 6 and SDF-1 have been shown to be involved in the onset and progression of OA (Goldring, 2007). Cytokines and chemokines bind to their respective receptors on chondrocytes to trigger intracellular kinase signaling, stimulate the synthesis of matrix metalloproteinase (MMP), and promote matrix degradation and cell migration (Borzì et al., 2000). It was thought that the pro-inflammatory cytokines and chemokines come from intra-articular tissues outside of cartilage, such as synovium, fat pad, blood vessels, bone periosteum, and bone marrow (Xie and Chen, 2019). Cartilage has not been considered as a source of inflammatory cytokines and chemokines because of its lack of immune cells and blood vessels that produce such inflammatory factors.

The most common type of cell in articular cartilage is the chondrocyte. In adult humans, normal healthy articular chondrocytes are quiescent (Goldring, 2012). The collagen fibrils comprising Col II, XI, and IX are synthesized and laid down by chondrocytes during development (Mendler et al., 1989). The half-life of collagen is more than 100 years (Sivan et al., 2008), which favors a stable extracellular matrix (ECM) that carries out its structural functions. Metabolism of the quiescent chondrocytes is activated by injury and/or inflammation during post-traumatic OA and aging-associated OA (Goldring, 2012). OA chondrocytes increase the synthesis of ECM molecules as well as ECM degradative enzymes (Goldring, 2000b). It was thought that cartilage ECM turnover and remodeling depend on the balance of anabolism and catabolism in chondrocytes (Ragan et al., 2000). The pro-inflammatory environment such as the elevation of cytokines and chemokines in the joint tilts the balance toward catabolism during OA.

It has been demonstrated that articular chondrocytes undergo cell senescence during aging and/or injury (Ming et al., 2018; Chu et al., 2020). The concept of cell senescence was first proposed by Hayflick almost 60 years ago (Hayflick and Moorhead, 1961). He showed that through serial cell passaging and subculture, fetal lung fibroblasts ceased dividing and reached senescence (replicative senescence) (Hayflick and Moorhead, 1961). Senescent cells express molecular markers of cell senescence including p16^*INK4a*^, erode telomeres, and acquire the senescence associated secretory phenotypes (SASPs) (Coppé et al., 2011). The SASP contains aforementioned pro-inflammatory cytokines and chemokines as well as matrix degradative enzymes including MMPs (Tchkonia et al., 2013). It has been shown that human OA cartilage contains senescent chondrocytes that exhibit all the hallmarks of cell senescence including p16^*INK4a*^ and SASP (Martin and Buckwalter, 2003). While it is well established that serial passage of human primary chondrocytes induces dedifferentiation into a fibroblastic phenotype, it is not clear whether it also induces replicative senescence (Edith et al., 2019).

For a very long period, it was thought that chondrocytes were the only cell type in cartilage. In 2004 mesenchymal stromal cells (MSCs), which were also considered to be a kind of chondroprogenitor cell, were found in normal and OA human articular cartilage (Alsalameh et al., 2004; Dowthwaite et al., 2004). These MSCs can be isolated through their adhesion to fibronectin on a culture plate while chondrocytes do not (Alsalameh et al., 2004; Dowthwaite et al., 2004). They are positive for CD166 while chondrocytes are CD166 negative (Liu et al., 2020). They have low abundance in normal young cartilage (1%), which is increased to 10% and more during OA pathogenesis (Alsalameh et al., 2004). However, it is not known how their abundance is increased and whether such increase contributes to OA pathogenesis. To answer these questions, we isolated MSCs from aging human OA cartilage (OA-MSC) (Jayasuriya et al., 2018) and from normal cartilage of young adults (NCSC) (Jayasuriya et al., 2019). In this study, we test the hypothesis that OA-MSCs are the senescent cells in cartilage whose abundance is increased during aging and OA pathogenesis. We provide molecular evidence that OA-MSCs are responsible for synthesizing the SASP that contributes to OA pathogenesis.

## Results

## Elevated Pro-inflammatory Protein Expression in OA-MSC

**FIGURE 1:**
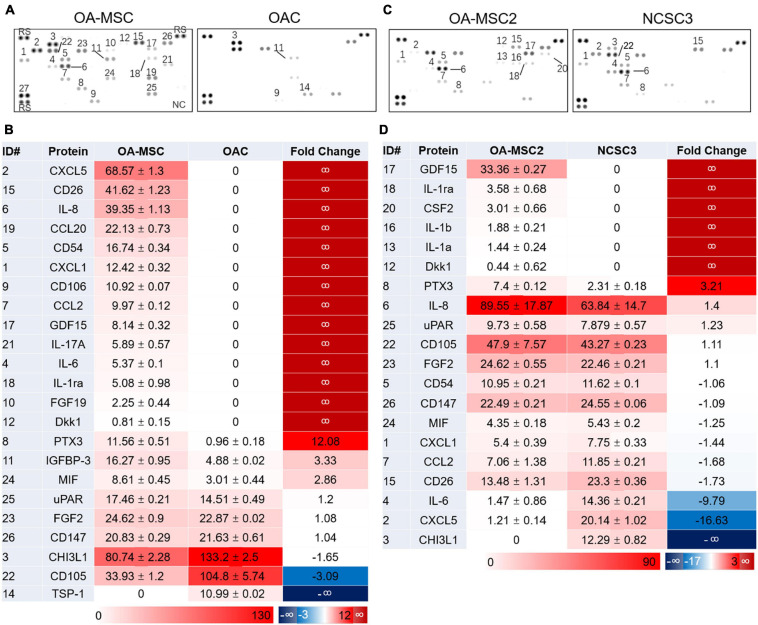
Human cytokine protein array analysis. Differential protein expression analysis of cytokines and related proteins was performed using cell extracts of primary human cartilage cells OA-MSC and OAC **(A,B)**, and cell extracts of human cartilage MSC cell lines OA-MSC2 and NCSC3 **(C,D)**. **(A,C)** Cytokine protein signals were detected on the membranes of protein arrays. ID number of the protein was indicated. **(B)** Differential protein expression profiles of OA-MSC and OAC. The unit number of the mean spot pixel density signal was indicated for each detected protein in OA-MSC (third column) and OAC (fourth column). Red color reflects the protein expression level. The relative fold change of protein signals of OA-MSC vs. OAC was indicated in the fifth column. Red color indicates higher protein levels in OA-MSC while blue color indicates higher protein levels in OAC. **(D)** Differential protein expression profiles of OA-MSC2 and NCSC3. The unit number of the mean spot pixel density signal was indicated for each detected protein in OA-MSC2 (third column) and NCSC3 (fourth column). Red color reflects the protein expression level. The relative fold change of protein signals of OA-MSC2 vs. NCSC3 was indicated in the fifth column. Red color indicates higher protein levels in OA-MSC2 while blue color indicates higher protein levels in NCSC3.

To determine the pro-inflammatory protein profiles in human OA cartilage cells, we isolated two types of cells from cartilage, OAC and OA-MSC, using fibronectin-adhesion assay as described previously (Fellows et al., 2017; Jayasuriya et al., 2018). Flow cytometry analysis confirmed that the fibronectin adherent cells were CD166+ OA-MSC, and the fibronectin non-adherent cells were CD166− OAC (Jayasuriya et al., 2018). Since human OAC was prone to de-differentiation after multiple passages, only early-passage of primary OAC and OA-MSC were used for protein array analysis. Among 105 cytokines, chemokines, and other pro-inflammatory factors on the panel (Supplementary Table 1), a total of 19 proteins were detected in OA-MSC and OAC (Figures 1A,B). Among them, 18 were detected in OA-MSC (94.7%) while 5 (22.7%) were detected in OAC; 16 were up-regulated in OA-MSC (84.2%) while 3 were up-regulated in OAC (15.8%) (Figure 1B). These observations indicated that OA-MSC was the predominant type of cell that expressed pro-inflammatory factors in cartilage in terms of the number of cytokines and the extent of their protein levels. The pro-inflammatory factors up-regulated in OA-MSC comprised major cytokines and chemokines implicated for OA pathogenesis including IL-6, IL-8, IL-1Ra, CXCL1, CXCL5, CCL20, and IL-17A (Figure 1B).

To determine whether the expression of pro-inflammatory proteins in OA-MSC was associated with aging, we compared the protein profiles of OA-MSC from older OA patients with NCSCs (normal cartilage stromal cells) from young non-OA patients. Since the NCSC cells comprised only 1% of total cartilage cells (Alsalameh et al., 2004), the number of primary NCSCs was too low to extract sufficient amount of protein required for proteomic analysis. To overcome this difficulty, we generated cell lines of NCSC (Jayasuriya et al., 2019) and OA-MSC (Jayasuriya et al., 2018) using primary CD166+ stromal cells isolated from normal and OA cartilage, respectively. An OA-MSC cell line (OA-MSC2) and a NCSC cell line (NCSC3) were randomly chosen for protein array analysis. Among 105 inflammatory factors on the panel (Supplementary Table 1), a total 16 proteins were detected in OA-MSC and NCSC (Figures 1C,D). Among them, 15 were detected in OA-MSC (93.8%) while 10 were detected in NCSC (62.5%); 9 were up-regulated in OA-MSC (56.3%) compared to NCSC while 7 were up-regulated in NCSC compared to OA-MSC (43.8%) (Figure 1D). Thus, the expression of pro-inflammatory factors of cartilage stromal cells was increased in the number of factors and the expression levels during aging process. The inflammatory cytokines up-regulated in OA-MSC than NCSC included IL-1α, IL-1β, and IL-8 (CXCL8), major SASPs that played important roles in OA pathogenesis (Coppe et al., 2010).

### Suppressed Chondrogenic Genes and Elevated SASP and Fibrosis Genes in OA-MSC

**FIGURE 2:**
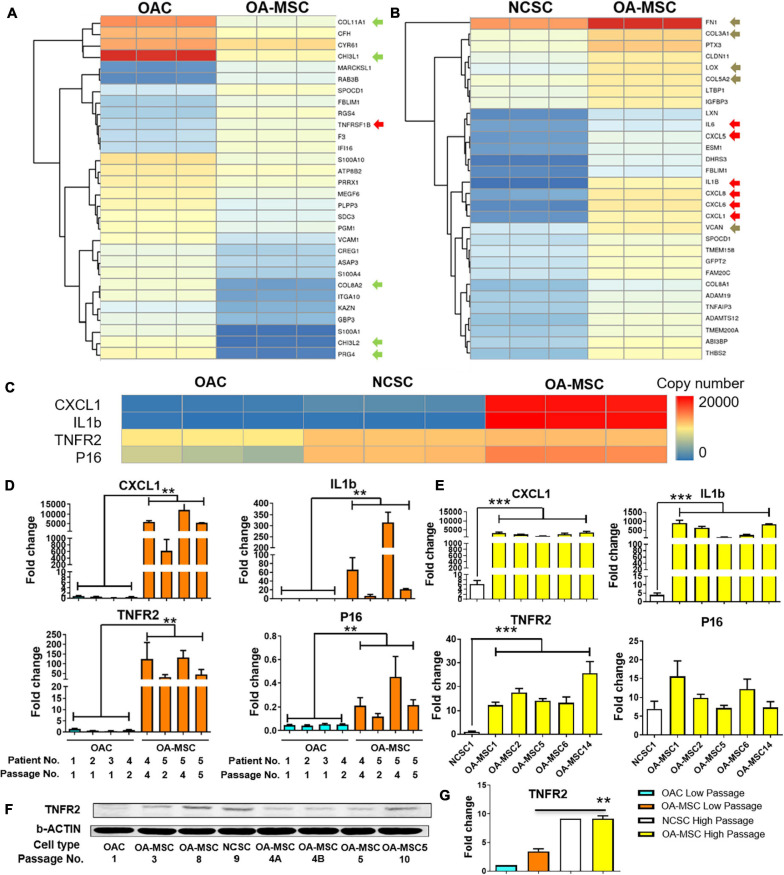
Differential gene expression analysis by RNA sequencing and validation by real-time PCR and western blot analysis. Heatmap of hierarchical clustering of top 30 most significant differentially expressed genes in OA-MSC vs. OAC **(A)**, and NCSC **(B)**. RNAseq data of OA-MSC2 and NCSC3 were utilized (*p* < 0.05). Green arrows: chondrogenic genes; red arrows: cytokine genes; brown arrows: fibrosis genes. **(C)** Heatmap of a group of highly expressed genes in OA-MSC in comparison to OAC and NCSC. The normalized transcript copy number of each gene was shown in triplicated RNA samples. RNAseq data of OA-MSC2 and NCSC3 were utilized (*p* < 0.05). Validation of RNAseq data by real-time RT-PCR analysis of the group of four highly expressed genes in OA-MSC **(D,E)**. **(D)** RNA samples were isolated from primary human OA articular cartilage cell cultures (OAC and OA-MSC) at different cell passages as indicated. The information of five OA patients is as follows. Patient 1: Female, Age 56 years; Patient 2: Female, Age 75 years; Patient 3: Female, Age 85 years; Patient 4: Female, Age 73 years; Patient 5: Male, Age 66 years. The value of each RNA sample group = mean ± SD. *N* = 3 for all groups. **p-value < 0.01. **(E)** RNA samples were isolated from cell culture of NCSC and OA-MSC cell lines. Real-time RT-PCR data of NCSC1 and five OA-MSC cell lines were utilized as indicated. The value of each RNA sample group = mean ± SD. *N* = 3 for all groups. ***p-value < 0.001. **(F)** Western Blot analysis of TNFR2 protein expression in OAC, OA-MSC and various NCSC and OA-MSC cell lines at different cell culture passages as indicated. The data shown is representative of three separate analyses. **(G)** Quantification of western blot analysis of TNFR2 protein levels in OAC, NCSC, and OA-MSC in low and high passage cell culture. The pixels of TNFR2/b-ACTIN were measured by software Image-Pro Plus. The relative TNFR2 protein expression level of OAC Lower Passage was set as 1. *T*-test was used for statistical analysis between OA-MSC Low Passage group and OA-MSC High Passage group. **p-value < 0.01.

To determine whether up-regulation of SASP protein expression occurs at the transcription level, we performed whole transcriptome RNA sequencing with the RNA purified from OAC, OA-MSC, and NCSC. Pairwise comparison was conducted with the 30 most significantly differentially expressed genes between OAC and OA-MSC (Figure 2A). Among them, 22 were down-regulated and 8 were up-regulated in OA-MSC in comparison to OAC. The RNAseq analysis indicated that the chondrocyte marker *CHI3L1* (chitinase-3-like-protein 1 or human cartilage gp-39) was the most significantly down-regulated genes in OA-MSC (Figure 2A, green arrow). This suggested that chondrogenic genes were suppressed in OA-MSC through down-regulation of their transcript levels. In addition, cartilage ECM markers including *COL11A1* and *PRG4* were up-regulated in OAC in comparison to OA-MSC (Figure 2A, green arrows). This indicated that OAC plays an anabolic role in synthesizing ECM in human OA cartilage. The up-regulated genes in OA-MSC included *TNFRSF1B* (*TNFR2*) (Figure 2A, red arrow), indicating that OA-MSC played a role in TNF-α signaling.

Pairwise comparison between NCSC and OA-MSC indicated that all the top 30 most significantly differentially expressed genes were up-regulated genes in OA-MSC (Figure 2B). This suggested a transcriptional activation during the cartilage MSC aging process. The most significantly up-regulated genes in OA-MSC were SASPs including *IL1B*, *IL6*, *CXCL1*, *CXCL5*, *CXCL6*, *CXCL8* (*IL-8*) (Figure 2B, red arrows). Since these are key inflammatory factors during OA pathogenesis, it suggested that OA-MSC play an important role in cartilage inflammation by synthesizing SASP. The mRNA levels of fibrosis markers in cartilage were also up-regulated in OA-MSC. They included fibroblastic *ECM FN1*, *COL3A1*, *COL5A2*, *VCAN*, and their crosslinking enzyme *LOX* (Figure 2B, brown arrows). This suggested that OA-MSC play an important role in cartilage fibrosis as well.

### SASPs Are Molecular Markers of OA-MSC

We focused on analyzing four molecules highly expressed by OA-MSC in the cell senescence and inflammation pathway: *CXCL1*, *IL1B*, *TNFR2*, and *p16*^*INK4a*^. RNAseq analysis indicated that two SASP molecules *CXCL1* and *IL1B* were highly expressed in OA-MSC (Figure 2C), suggesting that they were molecular markers of OA-MSC. *TNFR2* mRNA was expressed at a moderate level in OAC and its expression level became higher in NCSC and OA-MSC (Figure 2C). The expression of cell senescence marker *p16*^*INK4a*^ (*P16*) was low in OAC, higher in NCSC, and the highest in OA-MSC (Figure 2C). To validate the RNAseq data, we purified RNA in primary OAC and OA-MSC isolated from human cartilage of multiple patients (*n* = 5). Real-time RT-PCR analysis demonstrated that the *CXCL1* transcript was greatly up-regulated in OA-MSC than OAC (*p* < 0.01) (Figure 2D). Additional passages of primary OA-MSC in culture further increased the levels of *CXCL1* (Figure 2D). While *IL1B* mRNA was not detected in OAC, it was induced in OA-MSC (*p* < 0.01) (Figure 2D). Similarly, the mRNA levels of *TNFR2* (*p* < 0.01) and *P16* (*p* < 0.01) were significantly increased in OA-MSC than OAC, respectively. Passaging primary OA-MSC further increased the transcript levels of *IL1B*, *TNFR2*, and *P16*, similar to that of *CXCL1* (Figure 2D). These data suggest that the increase of the mRNA levels of the SASPs was associated with OA-MSC and its passages in culture.

To determine whether the increase of these OA-MSC markers was associated with aging, we compared their levels in the NCSC cells derived from a young patient and those of OA-MSC derived from old patients. The *CXCL1* and *IL1B* mRNA levels were greatly increased in OA-MSC compared to NCSC (*p* < 0.01) (Figure 2E). The mRNA levels of *TNFR2* (*p* < 0.01) and *P16* were increased in OA-MSC, respectively (Figure 2E). This suggested that the up-regulation of the OA-MSC markers was associated with the MSC aging process in cartilage. The increase of CXCL1 and IL1B expression in OA-MSC was also seen at the protein levels (Figure 1), and CXCL1 protein expression was also detected in NCSC. Western blot analysis demonstrated that, while the TNFR2 protein level was low in OAC, it was increased in OA-MSC especially at high cell passages (Figures 2F,G). Taken together the RNA and protein expression data, it suggested that the induction of SASP gene expression in OA-MSC occurred at both mRNA and protein levels.

### Cell Phenotype Transitions During Cell Aging and Senescence

**FIGURE 3:**
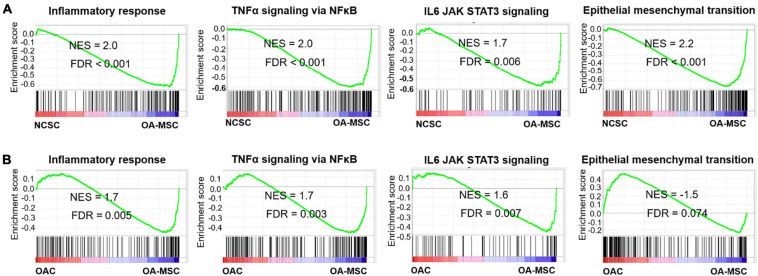
Gene Set Enrichment Analysis of key pathways activated during OA-MSC transition. **(A)** GSEA analysis indicated that inflammatory response (NES = 2.0, FDR < 0.001), TNFα signaling *via* NFκB (NES = 2.0, FDR < 0.001), IL6-JAK-STAT3 signaling (NES = 1.7, FDR = 0.006), and epithelial mesenchymal transition (EMT) (NES = 2.2, FDR < 0.001) were activated during the NCSC to OA-MSC transition. RNAseq data of human OA-MSC2 and NCSC3 were utilized for analysis. **(B)** GSEA analysis indicated that inflammatory response (NES = 1.7, FDR = 0.003), TNFα signaling *via* NFκB (NES = 1.7, FDR = 0.003), and IL6-JAK-STAT3 signaling (NES = 1.6, FDR = 0.007) were activated during the OAC to OA-MSC transition. RNAseq data of human OAC and OA-MSC2 were utilized for analysis. NES > 1.5 and FDR < 0.05 were considered significant.

To identify the pathways connecting OAC, OA-MSC, and NCSC, we performed Gene Set Enrichment Analysis (GSEA) (Subramanian et al., 2005) with the RNAseq data of these three types of cells. GSEA revealed multiple pro-inflammatory pathways were activated in OA-MSC in comparison to NCSC and OAC. They include inflammatory response (FDR < 0.001; FDR = 0.005), TNFα signaling via NFκ B (FDR < 0.001; FDR = 0.003), and IL6 JAK STAT3 signaling (FDR = 0.006; FDR = 0.007) (Figures 3A,B). This suggested that the acquisition of the pro-inflammatory phenotype in OA-MSC compared to NCSC and OAC. GSEA revealed that the ***epithelial mesenchymal transition*** (EMT) gene set was enriched during transition from NCSC to OA-MSC (FDR < 0.001) (Figure 3A), suggesting that the mesenchymal cell phenotype changes occurred during aging and senescence process.

### The OAC to OA-MSC Transition Involves Two Stages

**FIGURE 4:**
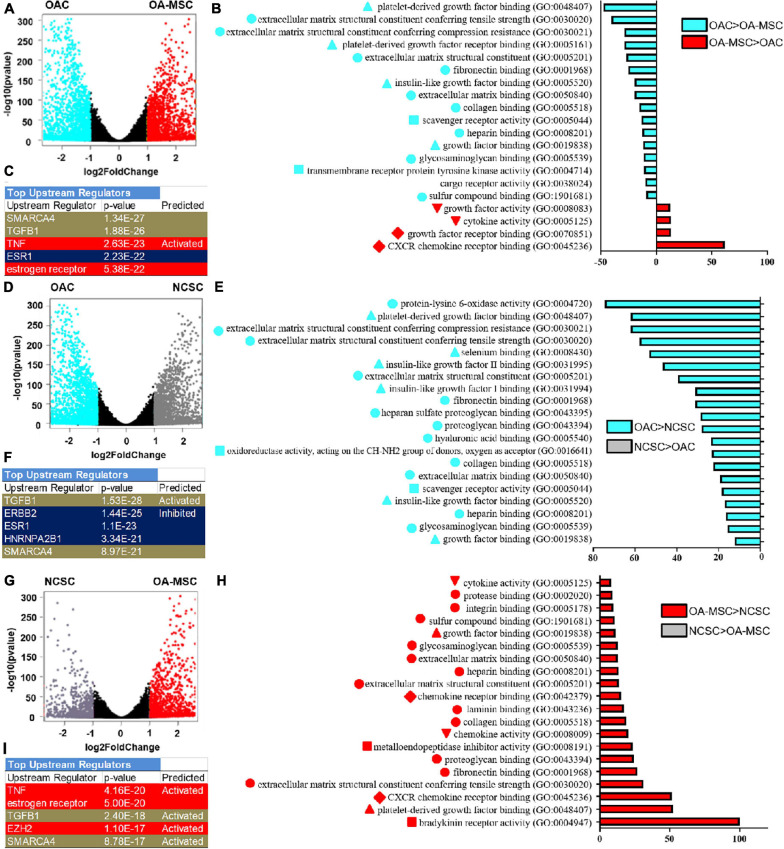
RNA sequencing bioinformatics analysis of OAC vs. OA-MSC **(A–C)**, OAC vs. NCSC **(D–F)**, and NCSC vs. OA-MSC **(G–I)** using volcano plot **(A,D,G)**, Gene Ontology (GO) **(B,E,H)**, and Ingenuity Pathway Analysis **(C,F,I)**. **(A)** Volcano plots analysis using RNAseq data of OAC and OA-MSC2 samples (*n* = 3). The points in aquamarine color means higher expressed genes in OAC while the points in red color means higher expressed genes in OA-MSC (fold change > 2, *p*-value < 0.05). Each point represents one gene. **(B)** GO classification of top twenty groups of genes based on RNAseq of OAC and OA-MSC2 samples (*n* = 3). Sixteen groups of genes (aquamarine color bars) were up-regulated in OAC. Classification with triangle: ECM regulatory function; classification with circle: ECM structural function; classification with square: cell receptor signaling; four groups of genes were up-regulated in OA-MSC (red color bars). Classification with inverted triangle: cytokine and growth factor activity; classification with diamond: chemokine and growth factor receptor binding. **(C)** IPA analysis of top upstream regulators based on RNAseq of OAC and OA-MSC2 samples (*n* = 3). Brown color indicates common regulators between OAC vs. NCSC and NCSC vs. OA-MSC. Red color indicates unique regulators of NCSC vs. OA-MSC. Blue color indicates unique regulators of OAC vs. NCSC. *p*-Values and predicted effects when known are indicated. **(D)** Volcano plots analysis using RNAseq data of OAC and NCSC3 samples (*n* = 3). The points in aquamarine color means higher expressed genes in OAC while the points in gray color means higher expressed genes in NCSC (fold change > 2, *p*-value < 0.05). Each point represents one gene. **(E)** GO classification of top twenty groups of genes based on RNAseq of OAC and NCSC3 samples (*n* = 3). All top up-regulated groups of genes were in OAC (aquamarine color bars). Classification with triangle: ECM regulatory function; classification with circle: ECM structural function; classification with square: cell receptor signaling. **(F)** IPA analysis of top upstream regulators based on RNAseq of OAC and NCSC3 samples (*n* = 3). Brown color indicates common regulators between OAC vs. NCSC and NCSC vs. OA-MSC. Blue color indicates unique regulators of OAC vs. NCSC. *p*-Values and predicted effects when known are indicated. **(G)** Volcano plots analysis using RNAseq data of NCSC3 and OA-MSC2 samples (*n* = 3). The points in gray color means higher expressed genes in NCSC and the points in red color means higher expressed genes in OA-MSC (fold change > 2, *p*-value < 0.05). Each point represents one gene. **(H)** GO classification of top 20 groups of genes based on RNAseq of NCSC3 and OA-MSC2 samples (*n* = 3). All top up-regulated groups of genes were in OA-MSC (red bars). Classification with triangle: ECM regulatory function; classification with circle: ECM structural function; classification with square: cell receptor signaling; classification with inverted triangle: cytokine and growth factor activity; classification with diamond: chemokine and growth factor receptor binding. **(I)** IPA analysis of top upstream regulators based on RNAseq of NCSC3 and OA-MSC2 samples (*n* = 3). Brown color indicates common regulators between OAC vs. NCSC and NCSC vs. OA-MSC. Red color indicates unique regulators of NCSC vs. OA-MSC. *p*-values and predicted effects when known are indicated.

To analyze cell transitions among OAC, NCSC, and OA-MSC, we performed volcano plot and Gene Ontology (GO) analysis. The volcano plot analysis demonstrated differential gene expressions in OAC and OA-MSC (Figure 4A). GO enrichment analysis indicated that, among the top 20 gene sets that were significantly differentially expressed between OAC and OA-MSC, 16 were up-regulated in OAC while 4 were up-regulated in OA-MSC (Figure 4B). The up-regulated gene sets in OAC consisted of three categories: (1) ECM structural function (ECM binding, structural constituent, and mechanical properties); (2) ECM regulatory function (growth factor binding); and (3) cell receptor signaling (scavenger and cargo receptor activity and transmembrane receptor tyrosine kinase activity) (Figure 4B, OAC > OA-MSC). The up-regulated gene sets in OA-MSC were (1) cytokine and growth factor activity, and (2) chemokine and growth factor receptor binding (Figure 4B, OA-MSC > OAC). Thus, the OAC to OA-MSC transition involved (1) down-regulation of chondrogenic ECM and receptor gene expression and (2) up-regulation of cytokine/chemokine and receptor binding.

RNA sequencing analysis suggested that down-regulation of chondrogenic ECM and receptor gene expression occurred during the OAC to NCSC transition (Figures 4D,E). Volcano plot indicated that more genes were significantly down-regulated in NCSC, suggesting a transcriptional repression during the transition of OAC to NCSC (Figure 4D). Among the down-regulated genes were chondrocyte markers *CHI3L1*, *COL11A1*, and *PRG4* (Supplementary Figure 1, green arrows), the same down-regulated genes during the OAC to OA-MSC transition (Figure 2A). Consistent with this finding, GO enrichment analysis indicated that all top 20 gene sets differentially expressed between OAC and NCSC were down-regulated in NCSC (Figure 4E). They contained the same three categories of gene sets down-regulated during the OAC to OA-MSC transition (Figures 4B,E).

### OA-MSC Acquires Inflammatory and Fibrosis Gene Expression During Cell Aging

RNA sequencing analysis suggested that up-regulation of cytokine/chemokine and receptor gene expression occurred during the NCSC to OA-MSC transition. The volcano plot analysis demonstrated that more genes were significantly up-regulated in OA-MSC than NCSC (Figure 4G), suggesting the OA-MSC transcriptome was activated during aging. GO enrichment analysis indicated that OA-MSC contained all top 20 up-regulated gene sets between NSCS and OA-MSC (Figure 4H). The up-regulated gene sets contained the same cytokine/chemokine gene sets upregulated during the OAC to OA-MSC transition (Figure 4B). In addition, the up-regulated gene sets also contained fibrotic ECM genes, consistent with the pair-wise RNAseq analysis (Figure 2B).

To identify the upstream regulators of the transitions among OAC, NCSC, and OA-MSC, we performed Ingenuity Pathway Analysis (IPA). The top five upstream regulators of the OAC to OA-MSC transition were (1) SMARCA4, a chromatin ATPase and transcription regulator; (2) TGFB1, a growth factor that activated catabolism in OA-MSC (Liu et al., 2020); (3) TNF, an inflammatory cytokine activating joint degeneration; (4) ESR1; and (5) estrogen receptor, critical regulators of estrogen signaling during OA pathogenesis (Figure 4C). These factors were involved in the OAC to NCSC transition (Figure 4F) and the NCSC to OA-MSC transition (Figure 4I). Two of these factors SMARCA4 and TGFB1 were common regulators. While ESR1 regulated the OAC to NCSC transition, estrogen receptor and TNF regulated the NCSC to OA-MSC transition. The unique regulators of the OAC to NCSC transition contained ERBB2 (a receptor protein kinase) and HNRNPA2B1 (a RNA binding and processing protein). TNF and EZH2 (a histone methyltransferase) were the top unique factors in the NCSC to OA-MSC transition, illustrating the role of inflammation and epigenetic regulation during MSC aging and senescence.

### Serial Passaging of OAC Induced Dedifferentiation and Recapitulated Cell Senescence and Inflammation Process

**FIGURE 5:**
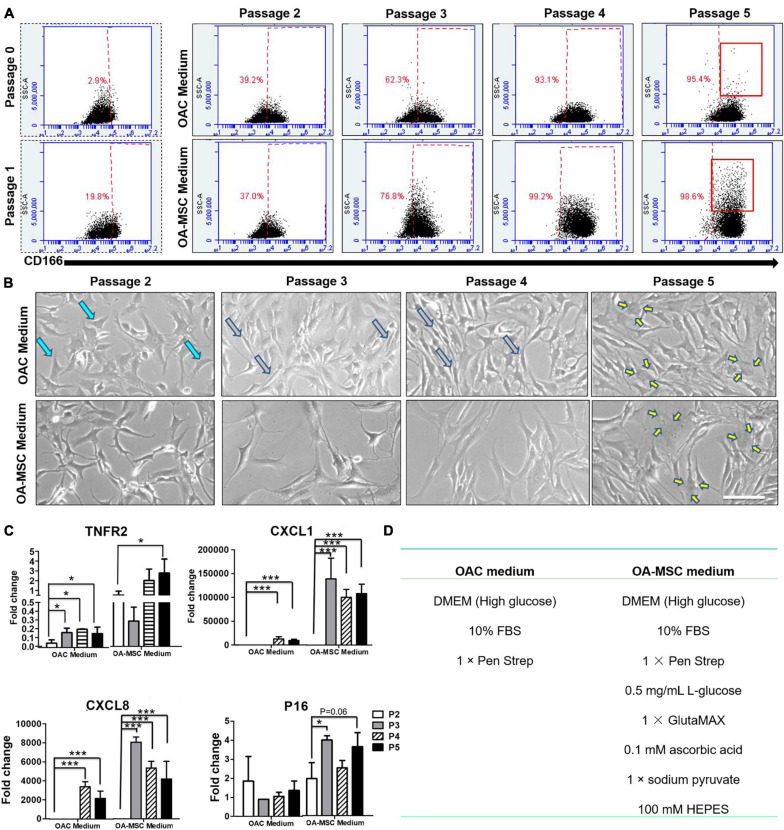
Serial cell culture passage of OAC induces dedifferentiation into NCSC-like cells and subsequent senescence into pro-inflammatory OA-MSC. **(A)** FACS analysis of CD166 positive cells during OA chondrocyte passaging in OA-MSC culture medium or OAC medium. The percentage of CD166 positive cells in each passage (*x*-axis) was indicated in red. The increase of the number of large sized cells (*y*-axis), a characteristic of senescent cells, was indicated by brackets in Passage 5. The data is representative of three experiments. **(B)** Cell morphology change of OA chondrocytes during cell culture passages in OAC medium and OA-MSC culture medium. OAC changed its morphology from cuboidal shape typical of chondrocytes (aquamarine arrows) in early passages (2 and 3) to spindle shape typical of fibroblasts (gray arrows) in mid passage (4) to large cells with processes typical of senescent cells (yellow arrows) in late passage (5). Bar = 200 μm. **(C)** Expression of aging related genes during OAC culture passages in OAC medium and OA-MSC medium. mRNA levels of TNFR2, CXCL1, CXCL8, P16 were quantified by real-time RT-PCR. The value of each sample group = mean ± SD. *N* = 3 for all groups. *p-value < 0.05; ***p-value < 0.001. **(D)** The components of OAC medium and OA-MSC medium.

To determine whether OAC could be transitioned into OA-MSC, we cultured primary articular chondrocytes from OA patients through serial passaging. OAC were cultured under standard conditions recommended for chondrocytes (OAC medium) or mesenchymal stromal cells (OA-MSC medium) (Figure 5D). Both OAC and OA-MSC media contained identical components including high glucose DMEM, 10% FBS, and antibiotics (Penn Strep). In addition, OA-MSC medium contained high energy sources including additional L-glucose, sodium pyruvate, and GlutaMAX. Flow cytometry analysis indicated that the percentage of CD166+ cells (MSC marker) increased during cell passagingg from the initial 2.9% at P0–95.4% at P5 (Figure 5A, *x*-axis). Cell morphology was also changed from the original cuboidal shape of chondrocytes (aquamarine arrows) to the long spindle shape typical of fibroblasts (gray arrows) during cell passages (Figure 5B). This fibroblast morphology was more evident during incubation in the OA-MSC medium. At P5, the senescent cell morphology (yellow arrows) of a large cell size with extended processes started to appear (Figure 5B). Flow cytometry confirmed more large-sized cells in the P5 culture (Figure 5A, *y*-axis). After P5, the cells reached full senescence and stopped proliferating. Flow cytometry also revealed that cells reached the CD166+ status faster when cultured in the OA-MSC medium (Figure 5A, *x*-axis).

Real-time RT-PCR analysis demonstrated that the markers of OA-MSC were induced by cell passaging (Figure 5C). The expression levels of these markers were higher when cells cultured in the OA-MSC medium than the OAC medium. Most interestingly, the pro-inflammatory cytokine/chemokine *CXCL1* and *CXCL8* (*IL-8*) were not detected at P2 in the OA-MSC medium or during P2–P3 in the OAC medium. This suggested that these cells acquired their inflammation phenotype at P3 in the OA-MSC medium and at P4 in the OAC medium. Taken together, these data suggested that the transition of OAC to OA-MSC was a gradual process comprising OAC dedifferentiation into NCSC-like MSC at P2 and acquiring the pro-inflammatory phenotype (SASP positive) at P4 in the OAC medium. In the OA-MSC medium, the pro-inflammatory phenotype (SASP positive) was acquired at P3. Thus, high glucose and other energy sources in the OA-MSC medium accelerated the transition from the non-inflammatory OAC phenotype to the pro-inflammatory OA-MSC phenotype associated with cell senescence.

### OAC, NCSC, and OA-MSC in Human OA Cartilage

**FIGURE 6:**
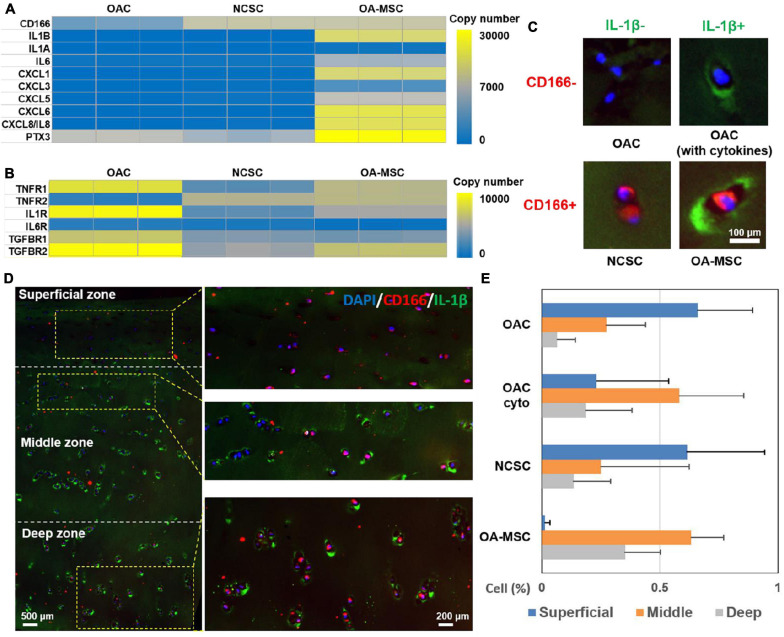
Identification of OAC, NCSC-like cells, and OA-MSC in human OA articular cartilage. **(A)** Differential expression analysis of cytokine, chemokine, and related genes in OAC, NCSC, and OA-MSC. RNAseq analysis was performed with OAC, NCSC3, and OA-MSC2 samples (*n* = 3). Yellow color represents higher copy number up to 10,000, while blue color represents lower copy number down to 0. Gray color represents intermediate copy number. Note that while IL-1β was synthesized by OA-MSC, neither OAC nor NCSC synthesized IL-1β. **(B)** Differential expression analysis of cytokine and chemokine receptors and related genes in OAC, NCSC, and OA-MSC. RNAseq analysis was performed with OAC, NCSC3, and OA-MSC2 samples (*n* = 3). Yellow color represents higher copy number up to 30,000, while blue color represents lower copy number down to 0. Gray color represents intermediate copy number. **(C)** Immunofluorescence analysis of different types of cells in human OA cartilage. While chondrocytes are CD166^–^, mesenchymal stromal cells are CD166^+^. OAC does not synthesize IL-1β but binds to IL-1β because it expresses IL-1R. OAC is CD166^–^ /IL-1β^–^; OAC binding to IL-1 β (OAC with cytokine) is CD166^–^ /IL-1β^+^, NCSC-like cell is CD166^+^/IL-1β^–^, and OA-MSC is CD166^+^/IL-1β^+^ cells in OA cartilage. CD166 was labeled by rhodamine red conjugated secondary antibody. IL-1β was labeled by green fluorescein conjugated secondary antibody. The nucleus was stained with blue Hoechst dye. **(D)** The double immunofluorescence histochemical analysis of CD166 and IL-1β in different zones in human OA articular cartilage. CD166, a marker of MSC, was indicated by rhodamine-conjugated secondary antibody (red). IL-1β, a marker of SASP was indicated by fluorescein-conjugated secondary antibody (green). The nucleus was stained with DAPI (blue). **(E)** The percentages of OAC, OAC (with cyto), NCSC-like cells, and OA-MSC in different zones of human OA articular cartilage (*n* = 3). Statistical analysis was presented in Supplementary Table 3.

To further define the roles of OAC, NCSC, and OA-MSC in cytokine and chemokine signaling, we analyzed the gene expression patterns of cytokines, chemokines, and their receptors (Figure 6). The RNAseq analysis showed that, while major cytokine and chemokines were synthesized by OA-MSC (Figure 6A), OAC contained the highest expression of the receptors of cytokines and chemokines, while OA-MSC contained the lower levels and NCSC contained the lowest levels of cytokine receptor expression (Figure 6B). This suggested that, while OA-MSC was the source of inflammation, OAC was a major target cell of inflammation signaling in cartilage.

To determine the distribution of OAC and OA-MSC in human OA articular cartilage, we performed double immunohistochemical analysis using antibody against CD166, a marker of MSC (Figure 6A), and IL-1β, a marker of SASP that was produced by OA-MSC (Figure 6A). Four types of cells were observed using these two markers: OAC (CD166−, IL-1β−), OAC with cytokine on cell surface (CD166− IL-1β+), NCSC (CD166+, IL-1β−), and OA-MSC (CD166+, IL-1β+) (Figure 6C). The CD166+ MSC cells in the superficial zone were negative for IL-1β, indicating they were NCSC (Figure 6D, superficial; Figure 6E, NCSC). In contrast, many CD166+ MSC cells were positive for IL-1β in the middle and deep zones, indicating these were OA-MSC (Figure 6D, middle, deep; Figure 6E, OA-MSC). While OAC were abundantly distributed in the superficial and middle zones, their content was reduced in the deep zone (Figure 6D, deep; Figure 6E, OAC). Interestingly, many OACs in the middle zone were IL-1β+ (Figure 6D, middle; Figure 6E, OAC cyto). This suggests that OAC is a recipient cell of cytokine signaling even though OAC does not synthesize IL-1β (Figure 6A).

## Discussion

Although OA was considered as a non-inflammatory disease, an ever-increasing body of evidence suggests that chronic degeneration of the joint is associated with persistent long-term low-grade inflammation in the joint (Jayasuriya et al., 2016). The source of inflammation in OA is unknown, although it has been shown to associate with high-fat diet, mechanical injury, and aging (Berenbaum, 2013). We have shown here that one of the sources of joint inflammation is OA-MSC within cartilage itself. OA-MSC synthesizes pro-inflammatory cytokines and chemokines that have been implicated in OA pathogenesis, including IL-1β, IL-6, IL-8, CXCL1, 5, and 6 (Goldring, 2007). We demonstrated that the induction of such pro-inflammatory molecules occurs at both mRNA and protein levels through genome-wide RNAseq and protein array analysis, respectively. We have also shown that the induction of inflammation in OA cartilage occurs during the transition from normal cartilage stromal cell (NCSC) in the young to the OA-MSC in the old during aging.

In recent years, cell senescence has been shown to be closely associated with OA pathogenesis (Ming et al., 2018; Chu et al., 2020). Injection of senescent cells into the joint space led to joint degeneration (Xu et al., 2017). Conversely, local clearance of p16^*INK4a*^-positive senescent cells from the joint attenuated injury and aging induced OA (Jeon et al., 2017). Although the role of senescent cells in causing joint degeneration has been established, the molecular mechanism by which a chondrocyte reaches senescence has not been well understood (Coryell et al., 2020). The expression levels of the cell senescence marker p16^INK4a^ and SASP were elevated in the serial passages of human chondrocyte culture *in vitro* and in aged human and mouse cartilage *in vivo* (Martin and Buckwalter, 2003). However, inactivation of p16^ INK4a^ in chondrocytes of adult mice failed to attenuate joint degeneration during aging or injury (Diekman et al., 2018). This observation raised an important question whether senescent chondrocytes were involved in cartilage degeneration.

We have shown here for the first time that OA-MSC, but not OA chondrocytes (OAC), has elevated levels of p16^*INK4a*^ and SASP. Therefore, OA-MSC, but not OAC, are the senescent cells that become a source of inflammation in the joint. Our study also provided a plausible molecular explanation to the observation that joint degeneration was not affected when the p16^*INK4a*^ gene was deleted in chondrocytes using the chondrocyte-specific Acan-Cre (Diekman et al., 2018). Since p16 is expressed at very low levels in the Acan-positive OAC (Figure 2C), targeting OAC for p16 knockout might not affect the real source of cell senescence in OA cartilage. Our data predict that joint degeneration would be attenuated if p16 were knocked out in OA-MSC, since it would abolish the source of SASP in OA cartilage. Although this prediction remains to be tested, OA-MSC should be considered as potential target cells of senolytics and anti-inflammation therapy for OA intervention in future studies.

**FIGURE 7:**
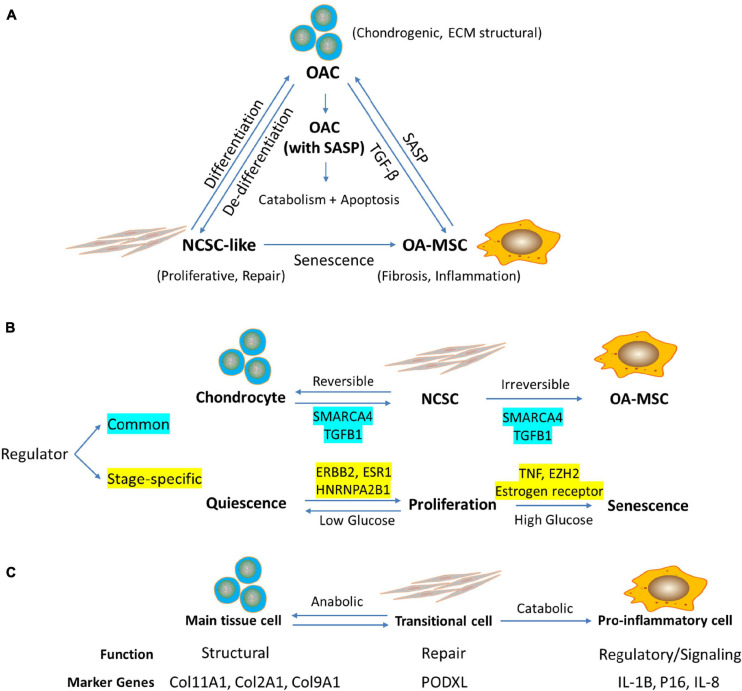
RNA sequencing analysis indicates the relationship among chondrocytes, mesenchymal stromal cells (MSC), and senescent MSC in human articular cartilage during aging. **(A)** Working hypothesis of the relationship among OAC, NCSC-like cells, and OA-MSC in aged human OA cartilage. Cartilage defect due to aging, stress, or injury induces proliferation and dedifferentiation of OAC, which results in NCSC-like cells for tissue repair. Repeated activation of NCSC-like cell replication in aged cartilage induces replicative senescence, which results in senescent OA-MSC. OA-MSC synthesizes pro-inflammatory SASP including IL-1b, IL-6, CXCL1, 5, 6, and 8. Upon binding its receptors on OAC, SASP activates cytokine-induced catabolism and apoptosis of OAC, thereby contributing to joint degeneration and OA pathogenesis. **(B)** Regulators of Senescence Associated Cell Transition (SACT) during cartilage aging. RNAseq analysis identified common and stage-specific regulators of cell transitions during chondrocyte aging process. The working hypothesis is that SACT comprises two cell transitions during aging. The first transition converts quiescent normal adult articular chondrocytes into proliferative and de-differentiated NCSC. The second transition converts proliferative NCSC into senescent and pro-inflammatory OA-MSC. The common regulators governing both transitions include SMARCA4 and TGFB1. The stage-specific regulators for the first transition include ERBB2, ESR1, and HNRNPA2B1. The specific regulators for the second transition include TNF, EZH2, and estrogen receptor. While the transition of chondrocytes to NCSC or vice versa is reversible, the transition of NCSC to OA-MSC may be irreversible. While high glucose drives MSC senescence and inflammation, low glucose may maintain the NCSC/chondrocyte balance for tissue repair (Jiang et al., 2016). **(C)** Proposed function and marker genes for OAC, NCSC, and OA-MSC in human articular cartilage. The working hypothesis is that chondrocytes serve a structural function as the main tissue resident cells in cartilage; NCSC serve as a transitional cell for tissue repair and wound healing in response to stress, injury, or aging; and OA-MSC serve as a regulatory, signaling cell during injury, and aging process. The marker genes for chondrocytes include chondrogenic ECM *COL11A1*, *COL2A1*, and *COL9A1*; NCSC marker gene includes MSC marker *PODXL*; and OA-MSC marker genes include cell senescence and SASP markers *IL1B*, *P16*, and *IL8*.

Although OA-MSC is a source of SASP in cartilage, the receptors of cytokines and chemokines are expressed by both OAC and OA-MSC. In particular, *IL-1R* and *TNFR1* are highly expressed by OAC. Although OAC does not synthesize IL-1β mRNA, IL-1β protein is associated with OAC cell surface in human OA cartilage. It suggests that OAC is a recipient cell for pro-inflammatory signals secreted by OA-MSC. On the other hand, TNFR2 is highly expressed by OA-MSC but only at the moderate levels in OAC. Since TNFR1 and TNFR2 may activate different pathways (Wajant and Siegmund, 2019), TNF-α may activate different cellular responses in OAC and OA-MSC. Past studies have shown that IL-1 binding to its receptor on chondrocytes induce high levels of MMP-13, a major catabolic proteinase responsible for collagen degradation (Goldring, 2000a). We suggest an inflammation signaling axis in OA cartilage in which OA-MSC produces pro-inflammatory signals while OAC receives inflammation signals and activates catabolism and apoptosis (Figure 7A). Even though OA-MSC is much fewer in number than OAC in cartilage, it may act as seed senescent cells to release SASP affecting surrounding OAC. OAC may serve as executing cells that amplify the biological responses triggered by the inflammation signals from OA-MSC. The interplay between OA-MSC and OAC *via* NCSC alters the balance of anabolic matrix synthesis and catabolic ECM degradation (Figure 7C).

Bioinformatics analysis of RNAseq data supports the concept of the interplay between OA-MSC and OAC. It indicates that the main function of OA-MSC is twofold: (1) production of SASP including cytokines and growth factors; and (2) binding to chemokines and growth factors for signaling that results in cell fibrosis and senescence. It also indicates that the main function of OAC is twofold: (1) production of chondrogenic anabolic ECM for structural and regulatory functions, and (2) receptor signaling in response to the microenvironment, which in turn modifies the surrounding matrix and neighboring cells. While both OA-MSC and OAC exist in the same aged OA cartilage, OA-MSC is senescent and OAC is not; OA-MSC is fibrotic and pro-inflammatory and OAC is chondrogenic and does not produce SASP. Thus, our data suggests that not all cells in an aged tissue are senescent. It is a specific type of cell, in this case senescent OA-MSC, which are responsible for the fibrotic and pro-inflammatory phenotype of an aged tissue. Since OA-MSC is much fewer in number than OAC, it suggests that cell senescence could be initiated in a few seed cells that become the foci of cell senescence in a degenerative tissue during aging.

Our study suggests that cell type transitions may occur during cell aging and senescence process (Figure 7B). They result in the interaction of different types of cells, which lead to cell death and tissue degeneration (Figure 7A). This process in termed “Senescence Associated Cell Transition and Interaction” (SACTAI). Testing the SACTAI concept will help our understanding of the cellular processes that lead to cell senescence, inflammation, and degradation. The GSEA bioinformatics analysis indicated that SACT is similar to epithelial-mesenchymal transition (EMT), although it would be more appropriate to term it chondro-mesenchymal transition (CMT) during cartilage aging and senescence. There are many similarities between SACT and EMT. First, like EMT, CMT involves attenuation of tissue specific chondrogenic gene expression, chondrocyte morphology and matrix adhesion, and a gain of migratory and invasive properties including secretion of pro-inflammatory cytokine, chemokine, and MMPs. Second, both CMT and EMT involve cell phenotype conversion from a specialized quiescent somatic cell to a mesenchymal stem cell capable of self-replicating and subsequent differentiation into different cell lineages. Third, EMT has been shown to occur not only during development and organogenesis but also in the physiological and pathological processes during adulthood including wound healing, tissue fibrosis, and cancer progression (Georgakopoulos-Soares et al., 2020). Since cell senescence has been shown to be involved in these events, SACTAI will likely play an important role not only during development but also in adult tissue homeostasis and aging.

Our study also suggests SACTAI may be induced by repeatedly activation of cell replication. Replicative senescence of MSC during joint aging is supported by the literature on MSCs harvested beneath the articular cartilage (Vozzi et al., 2016; Dicarlo et al., 2018), and MSCs from synovium (Dicarlo et al., 2019). In the original discovery of replicative cell senescence Hayflick and Moorhead (1961) isolated human fibroblasts from fetal lung and cultured to reach confluence. Cell proliferation was activated repeatedly in a series of cell passages in culture. After about 50 culture sub-cultivations, cells reached senescence before death. Subsequently it was discovered that senescent cells synthesized pro-inflammatory proteins termed SASPs (Coppé et al., 2011). In this study, we performed similar serial cell culture passages of aged osteoarthritic human chondrocytes to repeatedly activate cell proliferation. We found that it took as few as five cell passages to reach cell senescence. Chondrocyte replication in serial culture passages induced cell dedifferentiation and subsequent senescence. During de-differentiation of primary chondrocytes into fibroblastic MSCs, the chondrogenic phenotype of OAC was suppressed by transcriptional repression, resulting in NCSC-like MSCs. We also observed such NCSC-like MSCs (CD166+; IL-1β−) in human OA cartilage (Figure 6). Thus these transitional MSCs may exist *in vivo*. Such OAC-derived NCSC-like cells were also observed by other laboratories previously (Oda et al., 2016). Low glucose concentrations were shown to be critical for maintaining the regenerative capacities of these NCSC-like cells (Han et al., 2004). We show here that high glucose leads to accelerations of MSC senescence to pro-inflammatory OA-MSC (Figure 7B).

Our RNAseq analysis indicates that, during MSC senescence, the inflammatory and fibrotic phenotypes of OA-MSC were acquired by transcriptional activation. Serial passages of primary OAC not only decreased the percentage of chondrocytes but also increased the percentage of MSC, which ultimately resulted in senescent OA-MSC. A shortcoming of this study is that we did not determine the percentage of OA-MSC derived from cell type transition or proliferation. Such analysis requires performing *in vivo* cell lineage tracing studies in the future. Through immunohistochemistry analysis, we observed multiple cell types in human OA cartilage including chondrocytes (CD166−) and MSCs (CD166+) in both SASP positive and negative categories. Existence of these multiple types of cells in human OA cartilage not only suggests their interactions with each other spatially (Figure 7A), but also potential cell type transitions temporally (Figure 7B). Recent single-cell RNAseq analysis also supports the concept of multiple cell types including chondrocytes, MSC (chondroprogenitor cells), and derived cell types in human OA cartilage (Ji et al., 2019). Importantly, our study here identified not only the senescent cell type and its markers in human OA cartilage (Figure 7C), but also the critical regulators driving chondrocyte dedifferentiation and MSC senescence (Figure 7B). The pro-inflammatory cytokines and chemokines secreted by OA-MSC may play a critical role in regulating OA pathogenesis in the joint during aging.

## Materials and Methods

### Human Tissue

Human articular cartilage tissues were collected from OA patients (Male and Female, Age: 56–85 years old) who underwent total knee replacement (OA cartilage) and young adult cancer patient (Female, 18 years old) who underwent leg amputation (normal cartilage). The use of discarded human cartilage tissues were approved by the Institutional Review Board (IRB) of Rhode Island Hospital.

### Cell Isolation and Cultivation

The OA-MSCs and OA were isolated from the normal looking tissue area of the full thickness OA articular cartilage. Cartilage samples were rinsed three times with PBS plus 1% penicillin–streptomycin and then cut into small pieces. The cartilage fragments were digested by 2.0 mg/mL Pronase (Roche, Indianapolis, IN, United States) for 30 min at 37°C with shaking water bath. Then cartilage fragments were rinsed with PBS three times and treated with 1.0 mg/mL Collagenase I (Sigma-Aldrich, St. Louis, MO, United States) in DMEM overnight in 37°C shaking water bath. Cells with medium were strained through a nylon cell strainer (100 μm) (BD, Franklin Lakes, NJ, United States) and rinsed with DMEM three times. Cells were plated in fibronectin (10 μg/mL in PBS) coated dishes for 20 min at 37°C in cell incubator. After 20 min, the adherent cells were primary OA-MSC while non-adherent cells were collected as OA chondrocytes. The OA-MSCs were cultured in OA-MSC medium: DMEM with 10% FBS, 1% penicillin–streptomycin, 100 mM HEPES, 1% GlutaMax, 0.1 mM ascorbic acid, 0.1 mM sodium pyruvate, and 2.7 μM L-glucose. The OAC were cultured in OAC medium: DMEM with 10% FBS, 1% penicillin–streptomycin, 100 mM HEPES, unless noted otherwise.

Establishment and characterization of OA-MSC cell lines and normal cartilage stromal cell (NCSC) lines were described previously (Jayasuriya et al., 2018, 2019). The OA-MSC and NCSC cell lines were found to retain the gene expression pattern and multi-lineage differentiation potentials of primary cell counterpart, respectively (Jayasuriya et al., 2018, 2019). The cartilage-derived OA-MSC and NCSC cell lines retain the MSC marker expression pattern and multi-lineage differentiation potentials as the primary MSC (Jayasuriya et al., 2018, 2019; Hu et al., 2019; Liu et al., 2020). The primary OAC and OA-MSC were from the same cartilage tissue thus eliminating the systemic differences between different tissues or patients. While OA-MSC was from old OA cartilage, NCSC was from young normal cartilage. Thus, the difference of OA-MSC and NCSC represents cell senescence changes during aging.

### Human Cytokine Protein Array

We performed protein expression analysis of OA-MSC, NCSC, OAC, and primary OA-MSC using a Human XL Cytokine Array Kit (R&D system, Minneapolis, MN, United States). All the procedures and data analysis were performed following the manufacturer instructions. Total protein was extracted from 1 × 10^7^ cells using Lysis Buffer 17 (R&D system, Minneapolis, MN, United States) supplemented with 10 μg/mL Aprotinin (Tocris Bioscience, Minneapolis, MN, United States), 10 μg/mL Leupeptin (Tocris Bioscience, Minneapolis, MN, United States), and 10 μg/mL Pepstatin (Tocris Bioscience, Minneapolis, MN, United States). Each sample containing 200 μg protein in 1.5 mL Array Buffer 6 was run on each array (membrane) under incubation at 4°C overnight on a shaker. Then the membrane was washed three times with Wash Buffer for 10 min on a shaker. Twenty microliters of Detection Antibody Cocktail in 1.5 mL Array Buffer 4/6 was added to each membrane for 1 h incubation on a shaker. 1× Streptavidin-HRP was added for 30 min after three times washing with Wash Buffer. The membrane was incubated with 1.0 mL Chemi Reagent Mix for 1 min with plastic sheet protector. The liquid was then squeezed out by paper towels. The membranes were exposed by ChemiDoc MP (BioRad, Hercules, CA, United States) to generate the profile of spot pixel density image. The density signals of each spot were quantified by Image Pro Plus. The relative differences of sample signals were compared based on the standardized signal unit.

### RNA Purification and Sequencing

Total RNA was purified from cultured cells using Qiagen RNAeasy kit. RNA sequencing was performed by Genewiz, Inc. Sequence reads were trimmed to remove possible adapter sequences and nucleotides with poor quality using Trimmomatic v.0.36. The reads were then mapped to the Homo sapiens GRCh38 reference genome available on ENSEMBL using the STAR aligner. The RNA-seq aligner is executed using a splice aligner which detects splice junctions and incorporating them to help align the entire read sequences. BAM files were generated as a result of this step. Unique exon hit counts were calculated using Feature counts from the Subread package. The top 30 significantly changed genes (fold change > 2) were selected by the lowest *p*-values to create Bi-Clustering Heat Map.

### Volcano Plot

All the genes are plotted for the volcano plot and each point represents one gene. The *x*-axis represents Log2FoldChange while the *y*-axis represents Log10 of *p*-value. An adjusted *p*-value < 0.05 and a Log2FoldChange > 1 are indicated in OA-MSC, OAC, and NCSC by yellow dots, blue dots, and gray dots, respectively.

### Gene Ontology (GO) Analysis

PANTHER System was used for the GO analysis (Mi et al., 2019). The genes with *p*-value < 0.05 and fold change > 2 were selected for the GO analysis. The “GO molecular function complete” mode was selected to generate a list of related gene numbers for each classification. The top 20 significant classifications with gene numbers in each comparison were selected to form a bar chart.

### IPA Pathway Analysis

Qiagen IPA system was used for analysis. All the analysis was performed based on base mean > 300, *p*-value < 0.05, fold change > 2. The top analysis and pathway analysis were performed in OA-MSC vs. OAC, OA-MSC vs. NCSC, and NCSC vs. OAC. The top 20 activated or inhibited pathways were selected into a bar graph. The red, green, and blue bars represented activated pathway in OA-MSC, OAC, and NCSC, respectively. The deeper in color means higher activation. The *x*-axis represents for log10 of *p*-value.

### Gene Set Enrichment Analysis (GSEA)

The RNAseq data was analyzed by pre-rank GSEA v3 (GSEA REF^1^) using the MsigDB v6.1 gene sets.^2^ NES > 1.5 and FDR < 0.05 were considered significant.

### Real-Time PCR

Total messenger RNA was isolated from cells using a Qiagen RNeasy Kit (Qiagen, Hilden, Germany) according to instructions. Each sample of 500 ng RNA was reverse transcribed using the miScriptIIRT Kit (Qiagen, Hilden, Germany). Real-time PCR was performed with the SYBR Green PCR Master mix (Qiagen, Hilden, Germany) using the Bio-Rad CFX96 real-time PCR detection system (Bio-Rad, CA, United States). The forward and reverse primer sequences were listed in Supplementary Table 2. Ribosomal RNA 18 S was used as the reference gene for normalization. Relative gene expression level was determined by the 2^–Δ ΔCt^ method.

### Flow Cytometry

Pre-conjugated antibodies CD166-FITC and mouse-IgG-FITC were purchased from Miltenyi Biotec (Bergisch Gladbach, Germany). Cells were washed twice with PBS and detached using TrypLE Express (Life Technologies, Grand Island, NY, United States). Cells were re-suspended with DMEM plus 10% FBS. Cells were then centrifuged of 300 × *g* and re-suspended with PBS twice. Next, 1.0 × 10^6^ cells were re-suspended in 100 μL Flow buffer (PBS (pH 7.2) with 0.5% bovine serum albumin and 2 mM EDTA). Ten microliters pre-conjugated antibody or negative control was added to each resuspension, mixed, and incubated for 10 min at 4°C in the dark. Cells were rinsed three times with PBS and re-suspended in 500 mL Flow buffer. Accuri C6 Flow Cytometer (BD Biosciences, San Jose, CA, United States) was used for flow cytometry analysis.

### Immunohistochemistry

Human OA articular cartilage of full thickness (*n* = 3) was processed and sectioned as previously described (Liu et al., 2020). The sections were treated by 3% hydrogen peroxide in methanol for 30 min, and by 100 mg/mL hyaluronidase for 15 min. After blocking non-specific binding with 3% bovine serum albumin (BSA) in PBS, immunohistochemistry was performed with primary antibodies (mouse anti-CD166 Abcam, MA, United States, catalog number: ab233750), 1:100; rabbit anti-IL-1β (Santa Cruz Biotechnology, TX, United States, catalog number: sc-7884) at 4°C overnight. Sections were stained for 30 min with a red fluorescently labeled anti-mouse secondary antibody and/or a green fluorescently labeled anti-rabbit secondary antibody (Invitrogen, MA, United States). The VECTASHIELD Mounting Medium with DAPI was used for cell nuclear staining and section storage. The section was Images were acquired at 4× and 20× magnification using a Nikon Eclipse 90i Digital Imaging System.

### Western Blot Analysis

Cells were washed, lysed, and processed as previously described (Liu et al., 2020). Equal amount of proteins for each sample were separated by 10% SDS-polyacrylamide gel and then transferred to Nitrocellulose (NC) membrane for 70 min at 100 V. The membrane was blocked with 5% bovine serum albumin (BSA) in Tris-buffered saline-Tween 20 (0.1%) (TBS-T) for 1hr at room temperature. Rabbit anti-TNFR2 antibody (Abcam, MA, United States, ab109322) were used at 1:1000 dilution at 4°C overnight. β-Actin was detected using anti-rabbit antibody (Abcam, MA, United States, ab179467) as reference protein. Membrane was rinsed with TBS-T for 10 min, five times and incubated with 1:5000 goat anti-rabbit-Alexa Fluor 680 or donkey anti-mouse-Alexa Fluor 680 (Molecular Probes, MA, United States) for 1 h at room temperature. The blots were scanned using an Odyssey fluorescence scanner (LI-COR Biosciences, NE, United States).

### Statistics

Unpaired student’s *t*-test was used for gene expression analysis in each two groups, which represent mean values ± SD (error bars). *p*-Value < 0.05 were considered statistically significant.

## Data Availability Statement

The data presented in the study are deposited in the NCBI GEO repository, accession number: GSE176199.

## Ethics Statement

The studies involving human participants were reviewed and approved by Rhode Island Hospital IRB Committee. The patients/participants provided their written informed consent to participate in this study.

## Author Contributions

WL contributed to design of the study, acquisition, analysis and interpretation of the data, drafting the manuscript and revising the content, and approval of the final version. AB contributed to analysis and interpretation of the data, revising manuscript, and approval of the final version. MF contributed to acquisition, analysis and interpretation of the data, revising manuscript, and approval of the final version. YL contributed to acquisition, analysis and interpretation of the data, revising manuscript, and approval of the final version. JD contributed to acquisition of the data, revising manuscript, and approval of the final version. CJ contributed to providing experimental samples, revising manuscript, and approval of the final version. QC contributed to conception and design of the study, analysis and interpretation of the data, drafting the manuscript and revising the content, and approval of the final version. All authors contributed to the article and approved the submitted version.

## Conflict of Interest

CJ and QC have pending patent applications of generating OA-MSC cell lines. OA-MSC cell lines have been licensed to Applied Biological Materials Inc. The remaining authors declare that the research was conducted in the absence of any commercial or financial relationships that could be construed as a potential conflict of interest.

## Publisher’s Note

All claims expressed in this article are solely those of the authors and do not necessarily represent those of their affiliated organizations, or those of the publisher, the editors and the reviewers. Any product that may be evaluated in this article, or claim that may be made by its manufacturer, is not guaranteed or endorsed by the publisher.

## Footnotes

1
https://pubmed.ncbi.nlm.nih.gov/16199517/

2
https://www.ncbi.nlm.nih.gov/pmc/articles/PMC4707969/

## Abbreviations

OACs: osteoarthritic chondrocytes
NCSCs: normal cartilage stromal cells
OA-MSC: osteoarthritis-mesenchymal stromal cell
ECM: extracellular matrix
CHI3L1: chitinase-3-like-protein 1
RT-PCR: reverse transcriptase-polymerase chain reaction
SASP: senescence-associated secretory phenotype
RNAseq: RNA sequencing
GO: Gene Ontology
GSEA: Gene Set Enrichment Analysis
IPA: Ingenuity Pathway Analysis
EMT: epithelial-mesenchymal transition
CMT: chondro-mesenchymal transition
SACTAI: Senescence Associated Cell Transition and Interaction.
