# Supplementary material for: Senescent Tissue-Resident Mesenchymal Stromal Cells Are an Internal Source of Inflammation in Human Osteoarthritic Cartilage
Source: Front Cell Dev Biol. 2021 Sep 6;9:725071. doi: 10.3389/fcell.2021.725071 (PMC8450518; doi:10.3389/fcell.2021.725071)
Supplement: Supplementary file 1 [file Data_Sheet_1.docx]

**Figure S1.** Hierarchical clustering heatmap of top 30 significant differential genes between human OAC and NCSC. RNAseq data of OAC and NCSC3 were used for analysis (n=3). Green arrows indicate chondrogenic marker genes up-regulated in OAC. The RNAseq data of OAC, NCSC, and OA-MSC have been deposited and can be accessed as the GSE176199 study at:
<https://www.ncbi.nlm.nih.gov/geo/query/acc.cgi?acc=GSE176199>


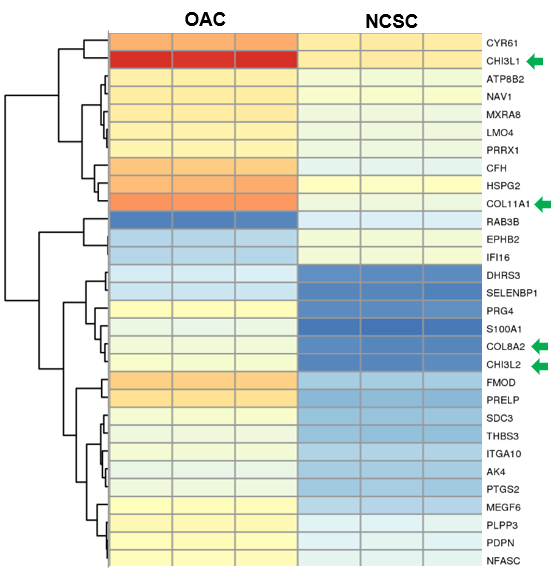


**Figure S2.** Heatmap of significant higher expression genes in NCSC compared with OAC and OA-MSC (baseMean > 300 and log2FoldChange > 2 in both OAC vs NCSC and OA-MSC vs NCSC). RNAseq data of OAC, NCSC3 and OA-MSC2 were used for analysis (n=3). Grey arrow indicates a MSC marker gene up-regulated in NCSC validated by real-time RT-PCR analysis.


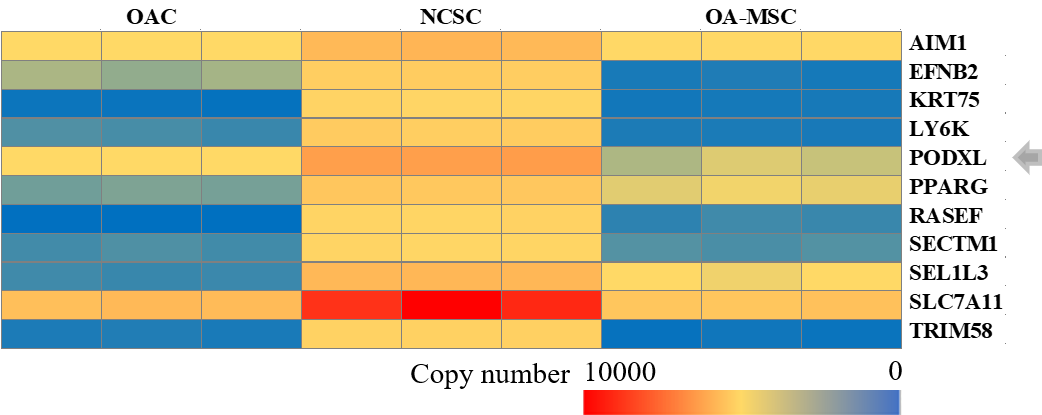


**Table S1.** The list of all 105 proteins in protein array.

**Table S2.** The forward and reverse primer sequences in real-time PCR reactions.

**Table S3.** One-way ANOVA analysis for Fig. 6E data. All four cell types showed significant difference in distributions across three zones (ANOVA p-value). In order to compare different zones, Tukey's test was performed. For example, OAC in superficial (S) zone is significantly different from middle (M) zone with a p-value of 0.0035.

|  | ANOVA | Tukey's test | | |
| --- | --- | --- | --- | --- |
|  | p-value | S vs. M | S vs. D | M vs. D |
| OAC | 7.7976E-05 | 0.0035 | < 0.0001 | 0.1186 |
| OAC cyto | 0.03745698 | 0.0833 | 0.9514 | 0.0476 |
| NCSC | 0.03640103 | 0.1232 | 0.0363 | 0.7876 |
| OA-MSC | 8.051E-07 | < 0.0001 | 0.0005 | 0.0025 |

S: superficial zone

M: middle zone

D: deep zone
